# Tracheotomy in Croup and Diphtheria, with Cases

**Published:** 1880-10

**Authors:** E. W. Lee, Christian Fenger

**Affiliations:** Surgeon to Cook County Hospital; Surgeon Pathologist to Cook County Hospital, Chicago


CHICAGO MEDICAL

JOURNAL & EXAMINER.

Vol. XLI.—OCTOBER, 1880.—No. 4.

### Original Communications.

### Article I.

Tracheotomy in Croup and Diphtheria, with Cases. By E. W. Lee, m.d., Surgeon to Cook County Hospital, and Christian Fenger, m.d., Surgeon Pathologist to Cook County Hospital, Chicago.

The main object in writing this paper and reporting these cases is to contribute our mite toward rescuing this operation from the doubtful position it occupies as a remedial measure and placing it in the position it merits.

Most authors in writing on this subject, mention the operation as one that may be done under certain circumstances and admit that lives possibly have been saved by it that otherwise would have been lost. To Trousseau belongs the credit of reviving and, in France, popularizing the operation. His unfailing faith through a series of years and the magnificent success attending his efforts are worthy of emulation for all time. It may be said that in his enthusiastic advocacy of it he resorted to the operation in many cases that would otherwise have recovered and hence his large average of successes. Admitting this, it proves the dangers following the operation are not so grave as had been supposed, and also that the earlier it is resorted to, the greater the chance of a successful issue. Vogel, in his work on Diseases of Children, says in the article on Croup (chapter 4, p. 259): "I have never yet seen a child recover from the genuine fibrinous croup, but from the diphtheritic form, three children out of twenty or twenty-five have recovered." Further on, p. 267, in speaking of tracheotomy as a remedial measure he says: "Let us assume that all the children operated on had genuine croup, the rate of recoveries (twenty-two per cent.), is nevertheless an extremely unfavorable one, and especially since the greater portion of the children operated on suffered from the milder diphtheritic forms." He also says he neither advocates or opposes the operation when proposed by other physicians.

Barclay in his article in Holmes' Surgery, on Croup and Diphtheria, says: "English medical men seem now very generally to incline to the opinion that the operation if not to be recommended, is at least justifiable, as it does not materially increase the risk of a fatal issue, and unquestionably in some cases, offers the only chance of recovery, *but to be successful it must be performed at an early period of the attack.* The italics are ours. Dr. E. H. Bennett, of Dublin, Ireland, says (in the Dublin *Journal of Medical Science*, March, 1880, p. 250), in relating a case where he had operated, "I make no apology for bringing forward this case, because most of the members know that tracheotomy for croup is rarely performed in Dublin, in consequence of the objections to the operation put forward by the late Mr. Porter, and the result of his strong objections to it has been that very few of us have had any experience of the operation." After alluding to Mr. Spence's experience, "ninety-five cases, recoveries one in three," he concludes: "Even with this condition of affairs I think we should set aside the opinion of Mr. Porter, and hold the operation to be admissible."

French surgeons, as a rule favor the operation, and in consequence of its timely performance have a high average of successful cases. In America the operation was was not much in vogue till the last decade. Meigs and Pepper, in 1870, Philadelphia, consistently advocated the performance of the operation in suitable cases, and in the Eastern cities it is now being practiced with a steadily increasing average of successful cases. Coming near home we have in this State, many earnest disciples of Trousseau, and judging from the success of their efforts, not unworthy ones either.

Dr. H. Z. Gill, of Jerseyville, Ill., in his report to the State Medical Society, gives a very elaborate exposition of the standing of the operation in our midst; he earnestly and consistently pushes its claim to recognition, and very pertinently remarks that out of the 200 or so deaths from croup and diphtheria in the city of Chicago, between forty and fifty might have been saved had the operation been resorted to. Drs. Bogue, Johnson, Andrews and others of Chicago, have been operating for several years, and, considering the desperate character of the cases, with a high average of successful results. In the concluding portion of Dr. Gill's report, he gives the opinion of some medical men in the State regarding the operation. One gentleman says he regards it as an unphilosophical irrational remedy, the results not justifying its performance. Another says a congress of physicians could not pursuade him to operate in diphtheria, but in croup he is always ready to be up and at it.

From this it will be seen that professional opinion is by no means a unit on the merits of the operation. The only way to settle the question is by practical experience. The man who condemns the operation and denies the patient the chance afforded by its performance assumes a far greater responsibility than he who operates on every available opportunity, and conscientiously carries out the after-treatment. We take the ground that as medical men, when all medical means have failed, we have no right to deny the little sufferer the last chance for its life, and furthermore that the parents have no moral right to do this either. In an experience extending over a number of years we can say that in no case where the operation has been advised and the advice rejected (and they have been many), has the child recovered. We trust the time is not far distant when the medical attendent will be as ready to perform tracheotomy as a life-measure, as he now is to adjust the obstetric forceps on an impacted head.

Within the past nine months we having been treating these cases (croup and diphtheria), by a continuous spray of chlorate of potash solution with one per cent. of carbolic acid, also lime-water carbolized to the same degree, and the success which has attended our efforts rather complicates than otherwise our selection of cases for operation.

Out of a series of between thirty and forty cases of all forms, there have been no less than eight recoveries where the larynx was involved, and which might, under ordinary circumstances, be considered suitable cases for operation. We do not claim that the treatment is new, but we do claim that it is more effectually carried out by means of large atomizers, so that the atmosphere of the apartment can be thoroughly saturated with the spray, and the little patient need not be alarmed by the proximity of the apparatus. The atomizer may best be placed in the center of the room, several feet away from the patient. After a careful trial of the various remedies used in the form of spray, we have come to the conclusion that lime-water with one per cent. of carbolic acid and four per cent. of glycerine possesses more solvent power over the membrane than any other. The grand question to be decided is when to operate. If we operate early, we have the satisfaction of saving a large percentage of cases, but feeling doubtful if some of these would not have recovered without surgical interference. If we operate late, we know by experience the rate of mortality rises to a discouraging height. So the first question that naturally arises is: Does the operation *per se* jeopardize the patient's life? We think to a slight extent it does.

As regards the actual performance of the operation, from our experience we are of the opinion, if due care be taken, the chances of mishap are so trifling as to cut but a small figure in the case. This may smack of temerity when we consider that Gross, that surgical veteran, says that he knows of no operation he approaches with greater dread than that of tracheotomy when the subject is a child with a short, fat neck. There is a dissimilarity in the difficulties attending this procedure which does not attend any other operation that we have been accustomed to perform. In Case 15, which was the child with the fat neck (and a truly difficult operation it proved), it seemed as if we never would reach the trachea; yet in Case 16, the patient about the same age and fully as robust, the trachea was exposed by a few strokes of the scalpel, and the operation completed with the utmost facility. The actual danger attending the operation commences at its completion. The presence of the tube and the direct entrance of air into the bronchii no doubt have an influence in producing those pulmonary complications which so often prove fatal, but their influence must not be over-estimated, as in many cases these complications are in existence before the operation has been attempted, the cause being the prolonged stridulous breathing, the operation not being resorted to in time. We will not stop now to describe the operation and the danger attending it, as it may be seen in any text-book on surgery. We may remark, however, *en passant*, that in opening the trachea we insert two tenaculæ, one each side of the site of incision. By this means we not only steady and lift the trachea, but by diverging the instrument at the point of insertion, we make the opening wide enough to admit the tube without difficulty. A very excellent device is to pass through the outer tube a closely fitting, soft rubber cathether, so that its point just projects beyond the end of the tube, the rubber being pointed, soft and pliable, readily pushes its way into the trachea without injuring the tissues. As regards the many instruments which have been devised for executing this maneuver, we would not recommend anyone to attempt the operation till he can dispense with their aid.

Martin, of Boston, has operated dispensing with the tube entirely. He cut down on the trachea in the usual manner, and by means of silk thread or silver wire passed through the trachea at the edge of the incision, he holds them apart. Another plan is to make a circular flap in the trachea and have it held back. These modifications we have not yet tried, but considering that the presence of a silver or rubber tube in the windpipe must be a source of irritation, they are deserving of a fair trial. Dr. Martin has had good success.

We now treat all cases of croup and diphtheria, whether there be laryngeal implication or not, by the continuous spray of lime-water with glycerine and carbolic acid in the proportions before mentioned.

We must digress somewhat in order to consider the subject from a pathological point of view. It is now beyond doubt that a sphero-bacterium—the so-called micrococcus diptheriens—is constantly found in the membrane of diphtheria and croup. Whether this minute organism is the product or bearer of diphtheric poison, or is only an unessential concomitant, finding the diphtheric infiltrated tissue the proper soil to thrive in, we do not know. Our knowledge about the character of the poison in contagious and miasmatic disorders is yet so defective that rational therapeutics based on anything but the blind *a posteriori* experience of olden times in most of these diseases has hitherto been an impossibility. Even if the micrococcus be no essential element of the diphtheric poison, it *is* an element, and the only one the presence of which we are sure of. It is rational, therefore, that we take this as a hint to a rational treatment, especially as most of the measures heretofore proposed have been abandoned because, after fair trial, they have proved to be useless. The first remedy to choose, of course, is some substance that will destroy the life and development of these micro-organisms—i. e., anti-bacteric remedies. As to the point of application of these remedies, it is natural to apply them to the air passages, as these parts are not only the main seat of the local disturbance of the disease, but most likely the parts through which the poison invades the body.

The inhalation of atomized fluid, as was said before, is nothing new, but we believe most of the older instruments were inefficient, and that antiseptic solutions for atomizing have not been used with the constancy and faithfulness they deserve.

Cases Nos 11 and 12 (Lee's) are fair examples in support of the treatment. Case 13 at first improved, but subsequently suffered a relapse. The chances for recovery would have been much better had the operation been resorted to at the first symptom of relapse. Cases 16 and 17 had been treated with the spray for several days before operation, and the condition of the membrane and the ease with which it was expelled, speaks volumes in favor of the treatment, as it must be recollected these were cases of genuine diphtheria where hitherto operative interference has been regarded as utterly futile. Where the evidences of blood poison are well marked, the laryngeal involvement and septic influence progressing *pari passu*, it is folly to suppose an operation could be of any benefit; where the characteristic listlessness is present, there is not the acute suffering for want of sufficient oxygen that exists in cases where the obstruction exists without the septic influence predominating; so it may be summed up that by the employment of antiseptic remedies, with the timely performance of tracheotomy in suitable cases, we give the patient a double chance. The antiseptic treatment *per se* is no doubt a powerful agent in our hands in battling with this dread disease, and in a certain number of desperate cases has proved an efficient remedy, but as we understand it we would not feel justified in trusting to it alone in cases grave enough to suggest operative interference.

With a view of testing the correctness of this theory, we would recommend a faithful and efficient application of the remedy, the patient having been placed under the spray, the internal medication consisting of quinine and alcohol in large and frequently repeated doses. And here we must again digress to protest against the employment of any depressing agent in treating these diseases. We are convinced that it is a septic poison generated in a congenial soil, and consequently must regard the use of emetics, escharotics, arterial sedatives, etc., as unphilosophical and irrational. If under the spray and internal stimulation the improvements be not steady and progressive, operative interference should be immediately resorted to, provided the nervous system be not overwhelmed by the septic poison. Every hour's delay enhances the danger, so quickly and surely the dreaded pulmonary complications are added to the previous difficulty. In Dr. E. H. Bennett's case, an examination made between ten and eleven o'clock in the forenoon failed to reveal any complication in the chest, yet in the evening there was a well-defined pneumonia, and at the time of the child's death—one o'clock the next day—the greater part of the lung had become absolutely solid. Remove the tube as early as possible (we commence on the fourth day), if only for an hour it can be dispensed with, a point is gained; next trial the child can breathe without its assistance for a longer period, and so on. The presence of the tube is a constant menace, and this fact should not be lost sight of. In Case No. 16 (Lee's) the child had pneumonia when operated on, and it was particularly desirable that the tube should be dispensed with as quickly as possible. At the end of the fourth day, Dr.

Lee removed it, and remained with the child for ten hours. Though at first the breathing was quite difficult, he refrained from re-introducing the tube, preferring to wait till it grew absolutely imperative. In a couple of hours the parts appeared to accommodate themselves to the altered condition, so that in twelve hours there was no further danger from that quarter.

M. Cairn, in the *St. Louis Courier of Medicine*, in reviewing the causes which may constitute an obstacle to the removal of the canula, mentions prominently two varieties of tracheal construction. The first variety, although rare, has, however, been demonstrated in a certain number of cases. It arises through the presence of fleshy growths springing from the wound, especially through those deeply seated upon the borders of the tracheal incision, and which grow in the midst of a cicatricial tissue projecting into the air passage after the closure of the cutaneous wound. The second variety up to the present time has not been described at all. A tracheotomized child was seized with a fit of suffocation just as the physician was attempting to effect a permanent removal of the canula. Examining the depths of the tracheal wound, he perceived a reddish prominence in the interior of the trachea which was taken for fleshy vegetation of the posterior wall. The child died in a fit of suffocation. Prof. Guyon recognized, upon the post mortem specimen sent him, that the projection regarded during life as vegetation was formed by the posterior wall of the trachea itself, which was folded longitudinally in its entire thickness. This folding was itself due to the approximation of the posterior extremities of the tracheal rings, separated anteriorly for the introduction of the canula. M. Currie, experimenting with the view of discovering the conditions of the production of this protrusion, concluded this particular variety of constriction which hitherto had not been pointed out, ought to be nevertheless rather frequent among children. It occurs after the introduction of the canula, and the more readily according as the membranous span which lies between the posterior extremities of the rings is large. It affects chiefly the first three rings of the trachea. The projection which results produces a tracheal constriction that may persist and prove a permanent obstacle to the removal of the canula.

| Name. | Age. | Cause. | Result. | Residence. | Operator. | Date. | Remarks. |
|---|---|---|---|---|---|---|---|
| 1. Nannie Keegan. | 3 years, 10 mos. | Diphtheria. | Died. | Chicago. | Dr. E. W. Lee. | Aug. 29, '77. | Sick three days. Used chloroform. Lived thirty hours. |
| 2. John Phalon. | 4 years. | Diphtheria. | Died. | Chicago. | Dr. E. W. Lee. | Oct. 18, '77. | Sick two days. Asphyxia imminent. Gave chloroform. Lived two and a half days. Died by asphyxia. |
| 3. Clara Nolan. | 7 years. | Memb. Croup. | Recovered. | Chicago. | Dr. E. W. Lee. | Oct. 23, '77. | Two weeks complaining. Chloroform. Artificial respiration necessary after the operation. Removed tube sixth day. |
| 4. Matthew Curran | 4 years. | Memb. Croup. | Died. | Chicago. | Dr. E. W. Lee. | Nov. 17, '77. | Sick thirty-six hours. Lived two and a half days. Died by exhaustion. Chloroform. |
| 5. Eddie Nolan. | 4 years. | Memb. Croup. | Died. | Chicago. | Dr. E. W. Lee. | Nov. 23, '77. | Sick five days. Chloroform. Lived twelve hours; died of exhaustion. |
| 6. Elice Purcell. | 3½ y'rs | Memb. Croup. | Died. | Chicago. | Dr. E. W. Lee. | Dec. 7, '77. | Sick four days. Lived four days. Died from asphyxia. Chloroform. |
| 7. Mich'l O'Rorque | 2 years, 2 mos. | Memb. Croup. | Recovered. | Chicago. | Dr. E. W. Lee. | March 18, '78. | Sick two days. Removed tube fourth day. Chloroform. |
| 8. James O'Grady. | 2 years, 4 mos. | Memb. Croup, no membrane visible. | Recovered. | 94 Brown Street, Chicago. | Dr. E. W. Lee. | Oct. 12, '78. | Three days sick. Tube worn nine days. Chloroform. Condition: Pulse, 140; respiration, 48; temperature, 103.5°; laryngeal obstruction permanent forty-eight hours; whispering voice; nasal dilation; retrocession of the base of the thorax marked; slight glandular enlargement. Low operation. Anæsthetic difficult of toleration. |
| 9. Albert Caproni. | 4 years. | Diphtheria. | Died. | 254 S. Halsted St., Chicago. | Dr. E. W. Lee. | Dec. 9, '78. | Four days sick. Died on fifth day after operation. Exhaustion from blood-poisoning. Chloroform well borne. Condition: Pulse, 130; respiration, 36; temperature, 10[illegible]°; laryngeal obstruction present forty-eight hours; tonsils soft; palate and fauces covered with a thick exudate; husky voice; dilated nostrils; epigastric sinking; glandular enlargement in neck. Low operation. |
| 10. Lucy Gray. | 3 years, 3 mos. | Cynanche Trachealis Croup. | Died. | 341 Fulton St., Chicago. | Dr. E. W. Lee, assisted by Drs. Van Buren, Bridge and Landis. | Dce. 27, '78, | Four days sick. Tube worn and lived 26 hours. Cause of death, exhaustion. Chloroform. Condition: "Asphyxiated, death imminent." Low operation. Parents grateful that the operation was done. Should have been done earlier. Diphtheria suspected. |
| 11. Lucas West. | 3 years, 3 mos. | Diphtheria. | Died. | S. Dearborn St., Chicago. | Dr. E. W. Lee. | Aug. 16, '79. | Lived sixty hours after operation. Immediate cause of death, asphyxia caused by extension of membrane. Anæsthetic, chloroform. Impending suffocation at time of operation. Low operation. |
| 12. Aaron West. | 5 years. | Diphtheria. | Recovered. | S. Dearborn St., Chicago. | Dr. E. W. Lee. | Aug. 17, '79. | Duration of previous illness, five days. Tube worn one week. Pulse, 160; respiration, 60; temperature, 103. Low operation. |

| NAME. | AGE. | CAUSE. | RESULT. | RESIDENCE. | OPERATOR. | DATE. | REMARKS. |
|---|---|---|---|---|---|---|---|
| 13. Norah Ryan. | 2 years. | Memb. Croup. | Died. | 48 Gurley Street, Chicago. | Dr. E. W. Lee. | Dec. 27, '79. | Duration of previous illness, one week. Lived fourteen hours after operation. Immediate cause of death—exhaustion. Anæsthetic, chloroform. Pulse, 168; temperature, 103.5°. Low operation. |
| 14. W. Madden. | 3 years. | Diphtheria. | Died. | Eighteenth St., Chicago. | Dr. Lee, assisted by Dr. Guerin. | Jan. 1, '80. | Duration of previous illness three days. Lived twenty-four hours after the operation. Immediate cause of death, asphyxia. Anæsthetic, none. Pulseless, deeply cyanosed. Respiration almost ceased. Low operation rapidly performed. |
| 15. Alice Green. | 3 years, 3 mos. | Diphtheria. | Died. | Blue Island, Ill. | Dr. Lee assisted by Drs. Herman and Kaufman. | Jan. 19, '80. | Previous illness, eight days. Lived six days. Cause of death, pneumonia. Anæsthetic, chloroform. Pulse, 144; respiration, 48; temperature, 103°. Low operation. |
| 16. C. J. Byrne. | 3 years, 6 mos. | Diphtheria. | Recovered. | W. Randolph St., Chicago. | Dr. Lee, assisted by Drs. W. E. Clark, Norman Bridge and son of Dr. Byrne. | Feb. 5, '80. | Previous illness, seven days. Tube worn seven days. Anæsthetic, chloroform. Pulse, 144; respiration, 64; temperature, 101°. Exudation of membrane over the soft parts of pteta and tonsils. Low operation. |
| 17. H. Graft. | 7 years. | Diphtheria. | Recovered. | 166 Brown St., Chicago. | Drs. Lee, assisted by Drs. McLennan and Leneman. | April 20, '80. | Previously ill, ten days. Tube worn five days. Anæsthetic, chloroform. At time of the operation: Pulse, 132; respiration, 48; temperature, 102°. Head thrown back, lips blue, face pale; epigastric retrogression. Two hours after operation: Pulse, 112 temperature, 101°; respiration, 36. Breathing with perfect freedom. High operation. |

| NAME. | AGE. | CAUSE. | RESULT. | RESIDENCE. | OPERATOR. | DATE. | REMARKS. |
|---|---|---|---|---|---|---|---|
| 1. Einzvahl. | 3 years. | Memb. Croup. | Recovered. | West Indiana street, Chicago. | Dr. Christian Fenger. Surg. to Cook County Hospital. | Oct. 18, '78. | Previous illness, three days. Anæsthetic, none. Dyspnœa urgent. Pulse and temperature high. Tube worn five days. |
| 2. F. Gehrig. | 2 years. | Diphtheria. | Died. | Chicago. | Dr. Fenger. | Oct. 25, '78. | Previous illness, ten days. Died Oct. 26. Immediate cause of death, "collapse." Condition at the time of operation, "collapsed." High operation. |
| 3. H. Kenzie. | 8 years. | Diphtheria. | Died. | Penn street, Chicago. | Dr. Fenger. | Sept 30, '79. | Previous illness, eight days. Lived four hours after the operation. Immediate cause of death, collapse, hæmorrhage. Severe dyspnœa, extensive diphtheritic exudation. Marked symptoms of blood-poisoning. Anæsthetic, chloroform. |
| 4. William Archer. | 3 years. | Diphtheria. | Died. | Chicago. | Dr. Fenger. | Oct. 27, '79. | Previous illness ten days. Lived twenty hours. Immediate cause of death, exhaustion. Anæsthetic, none. Diphtheritic exudation on tonsils, soft palate and extending into the larynx. Pulse rapid and feeble. Blood-poisoning. High operation. |
| 5. O. Malkon. | 4 years. | Diphtheria. | Recovered. | Chicago. | Dr. Fenger. | Dec. 21, '79. | Previous illness, seven days. Tube worn fourteen days. Anæsthetic, none. Tonsils and soft palate covered with diphtheritic exudation. Pulse and temperature high. High operation. |

